# Mathematical epidemiology is not an oxymoron

**DOI:** 10.1186/1471-2458-9-S1-S2

**Published:** 2009-11-18

**Authors:** Fred Brauer

**Affiliations:** 1 Department of Mathematics, University of British Columbia, Vancouver, BC, V6T 1Z2, Canada

## Abstract

A brief description of the importance of communicable diseases in history and the development of mathematical modelling of disease transmission is given. This includes reasons for mathematical modelling, the history of mathematical modelling from the foundations laid in the late nineteenth century to the present, some of the accomplishments of mathematical modelling, and some challenges for the future. Our purpose is to demonstrate the importance of mathematical modelling for the understanding and management of infectious disease transmission.

## Introduction

Communicable diseases such as measles, influenza or tuberculosis are a fact of modern life. Some diseases, such as chicken pox, usually have mild symptoms and vanish of their own accord. Others, such as Ebola (recurrently) and SARS, have appeared, causing a significant number of deaths, and then disappeared, but not before giving rise to fears of catastrophic spread. The prevalence and effects of many diseases in resource-constrained countries are probably less well-known but may be of even more importance. Every year, millions of people die of measles, respiratory infections, diarrhea and other diseases that are easily treated and not considered dangerous in the Western world. Diseases such as malaria, typhus, cholera, schistosomiasis and sleeping sickness are endemic in many parts of the world. The effects of high disease mortality on mean lifespans, and of disease debilitation and mortality on the economy in afflicted countries are considerable. A case in point is the AIDS epidemic, which has devastated life in much of Africa.

For some diseases, there are management methods, which may involve prevention (such as vaccination) or treatment of symptomatic patients. For diseases with no known treatment, it is possible to attempt control by isolation of diagnosed patients and quarantine of suspected victims to decrease transmission. However, it is not possible to do experiments to compare possible management strategies; the only way to attempt to compare the effectiveness of different approaches may be to formulate a mathematical model and use it to make predictions. Currently, HIV/AIDS causes many deaths and is a significant aspect in the social and economic structure of some countries, notably in Africa. Because of the long time scale on which HIV runs, a clinical trial to compare control strategies would take many years for results to be obtained. In order to provide useful information, models for the transmission of infectious diseases must be quantitative; this points to a need to develop mathematical models.

There have been many advances in disease management that have come from mathematical modelling. Two of the most striking are the recognition that mosquito management is the key to malaria control in a region and the realization that smallpox could be eradicated. However, there are many others.

## Communicable diseases in history

Throughout the course of history, communicable diseases have had major effects on human development. The Book of Exodus describes the plagues that Moses brought down upon Egypt; there are many other biblical references to diseases as historical influences, such as the decision of Sennacherib, the king of Assyria, to abandon his attempt to capture Jerusalem about 700 BC because of the illness of his soldiers (Isaiah 37,36-38). The fall of empires has been attributed directly or indirectly to epidemic diseases. In the second century AD, the so-called Antonine plagues (possibly measles and smallpox) invaded the Roman Empire, causing drastic population reductions and economic hardships, leading to disintegration of the empire because of disorganization, which facilitated invasions of barbarians. The Han empire in China collapsed in the third century AD after a very similar sequence of events. The population of China decreased from 123,000,000 to 65,000,000 around 1200 AD because of a combination of war with the Mongols and plague.

The defeat of a population of millions of Aztecs by Cortez and his 600 followers in 1519 can be explained, in part, by a smallpox epidemic that devastated the Aztecs but had almost no effect on the invading Spaniards, thanks to their built-in immunities. The Aztecs were not only weakened by disease but also confounded by what they interpreted as a divine force favouring the invaders. Smallpox then spread southward to the Incas in Peru and was an important factor in the success of Pizarro's invasion a few years later in 1532. Smallpox was followed by other diseases, such as measles and diphtheria, that were imported from Europe to North America. In some regions, the indigenous populations were reduced to one tenth of their previous levels by these diseases; between 1519 and 1530 the native population of Mexico was reduced from 30 million to 3 million.

The Black Death (bubonic plague) spread from Asia throughout Europe in several waves during the fourteenth century, beginning in 1346, and is estimated to have caused the deaths of as much as one-third of the population of Europe between 1346 and 1350. The disease recurred regularly in various parts of Europe for more than 300 years, notably as the Great Plague of London (1665-1666). It then gradually withdrew from Europe. As the plague struck some regions harshly while avoiding others, it had a profound effect on political and economic developments in medieval times. In the last bubonic plague epidemic in France (1720-1722), half the population of Marseilles, 60 percent of the population in nearby Toulon, 44 percent of the population of Arles and 30 percent of the population of Aix and Avignon died, but the epidemic did not spread beyond Provence.

The first attempt to construct the Panama Canal (1881-88) had to be abandoned because of yellow fever and malaria; the second attempt, beginning in 1907, was successful because of eradication of the mosquitoes which acted as vectors to spread these diseases.

Current concerns about a possible influenza pandemic are magnified by knowledge of the 1918 influenza pandemic, which caused a number of deaths estimated as between 50,000,000 and 100,000,000.

The historian W.H. McNeill argues, especially in his book [1], that the spread of communicable diseases frequently has been an important influence in history. For example, there was a sharp population increase throughout the world in the 18th century; the population of China increased from 150 million in 1760 to 313 million in 1794 and the population of Europe increased from 118 million in 1700 to 187 million in 1800. There were many factors involved in this increase, including changes in marriage age and technological improvements leading to increased food supplies, but these factors are not sufficient to explain the increase. Demographic studies indicate that a satisfactory explanation requires recognition of a decrease in the mortality caused by periodic epidemic infections. This decrease came about partly through improvements in medicine, but a more important influence was probably the fact that more people developed immunities against infection as increased travel intensified the circulation and co-circulation of diseases.

While epidemics may cause many deaths in a short time before disappearing, new diseases may also appear and become endemic. The appearance of AIDS about 1981, apparently initially in the population of men having sex with men in San Francisco, was such an event. It was identified as an immune disorder in the blood which could be spread in many ways: through sexual contact, shared needles for drug injections, transfusions with infected blood and by vertical transmission from mother to unborn child. It took two years for scientists to identify a virus, which became known as HIV, linked to AIDS. In 1987, a new drug (AZT) was developed which delayed progression of the disease, but many patients developed drug resistance. New drugs and combinations of drugs renewed hope, but they offered only improved length and quality of life, not a cure. It is estimated that in 2007 there were more than two million deaths from AIDS worldwide and more than 33, 000, 000 persons living with AIDS, including 330, 000 children. More than three quarters of these deaths were in sub-Saharan Africa. It is not an exaggeration to say that HIV/AIDS is the most urgent health problem for the entire world. While AIDS treatment rates have increased in some parts of the world, they remain very low in Africa and resource-poor countries.

Descriptions of epidemics in ancient and medieval times frequently used the term "plague" because of a general belief that epidemics represented divine retribution for sinful living. This view has not disappeared entirely. Some have described AIDS as punishment for sinful activities and such views have delayed or hampered attempts to control this modern epidemic.

In view of the importance of communicable diseases in history, it is natural that people would make efforts to understand the causes of diseases and search for treatments. This search leads naturally to an effort to construct models that focus on the main properties of a disease without necessarily attempting to include all the details.

## Why should we model?

**Figure 1:**
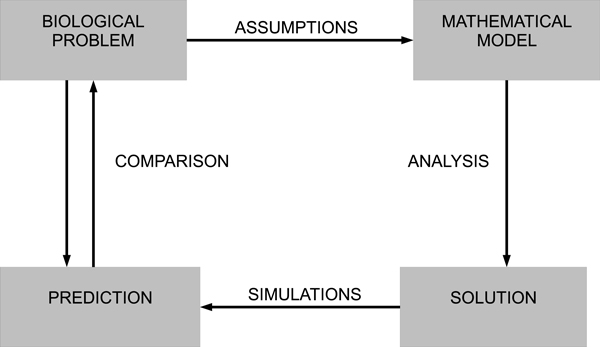
**Problems and Models**. A "flow chart" describing the relationship between scientific problems and models.

A model is an attempt to answer a question that begins with "Why?" The relation between problems and models in science may be described by the "flow chart" in Figure 1 (adapted from a similar flowchart in [2], by permission).

The normal process of scientific progress is to observe a phenomenon, hypothesize an explanation and then devise an experiment to test the hypothesis. A mathematical model is a mathematical description of the situation based on the hypotheses and the solution of the model gives conclusions which may be compared with experimental results. This comparison usually requires numerical simulations to give predictions which may be compared with observed data.

It has been observed in many epidemics that the disease spreads into a population and then disappears without infecting the entire population. Intuitively, one might think that epidemics die out because there are no people left in the location of the epidemic to be infected, but there is much evidence to contradict this explanation. In order to explain why part of the population escapes infection during an epidemic, it is natural to try to give a description of how the disease spreads. Such a description, or model, does not necessarily try to include all the details of the epidemic spread, but attempts to incorporate the factors that appear to be the most important. While a model may be a description in words, in order to compare observed results with a model prediction it is necessary to formulate the model mathematically. The general process is to make some assumptions about the way in which the epidemic spreads, formulate these assumptions in mathematical terms and translate them into a mathematical problem. This mathematical problem is a model of the epidemic.

For example, Kermack and McKendrick set out to try to explain why epidemics pass through a population without affecting the entire population [3]. Their mathematical model assumed mass-action incidence to describe the acquisition of infection followed by a period of infectivity and then recovery with immunity against reinfection. The simplest mathematical formulation of these assumptions is a pair of ordinary differential equations for the number of susceptible (uninfected) and the number of infected (and infectious) members of the population. They were able to describe the solution of this mathematical problem qualitatively, in terms of a quantity called the basic reproduction number that may be calculated in terms of the parameters of the model. This mathematical solution leads to the prediction that if the basic reproduction number is less than one, the number of infectives will tend to zero; if the basic reproduction number exceeds one, the number of infectives will increase initially before tending to zero, while the number of susceptibles decreases but never reaches zero. This prediction not only matches observations but also gives a criterion for whether a disease outbreak will develop into an epidemic or die out. In order to make more detailed predictions about the number of people infected in an epidemic, it would be necessary to make more detailed assumptions about the situation to give a more complicated mathematical model. Such a model would probably be sufficiently complicated that an exact solution would be impossible; numerical simulations would be needed to obtain predictions that could be compared with observations. Scientific experiments are usually designed to obtain information and to test hypotheses. For example, we might wish to compare two different management strategies for a disease outbreak. Experiments in epidemiology with controls are often difficult or impossible to design; even if it is possible to arrange an experiment, there are serious ethical questions involved in withholding treatment from a control group. In order to describe the course of a future disease outbreak, formulation and analysis of a mathematical model may be the only way to compare the effect of different management strategies. Mathematical modelling in epidemiology provides understanding of the underlying mechanisms that influence the spread of disease and, in the process, it suggests control strategies. In fact, models often identify behaviours that are unclear in experimental data. This may occur because data are non-reproducible and the number of data points is limited and subject to errors in measurement.

In the mathematical modelling of disease transmission, as in most other areas of mathematical modelling, there is always a trade-off between simple models, which omit most details and are designed only to highlight general qualitative behavior, and detailed models, usually designed for specific situations including short-term quantitative predictions. There is a tendency on the part of mathematicians to want to study models which are too simple to capture the essential properties of a disease, while there is a tendency on the part of epidemiologists to want complete models which may be too difficult to analyze properly. Detailed models are generally difficult or impossible to solve analytically; hence, their usefulness for theoretical purposes is limited, although their strategic value may be high. Also, more detailed models contain more parameters and therefore it may be more difficult to fit parameters to the model. Parameter fitting to a complicated model is especially dubious if data are sparse or of questionable accuracy. In order to be useful, a disease model should have a level of complexity appropriate to the amount of known information and the results desired. Ideally, a model should also have a level of complexity appropriate to the specific questions the model is designed to answer; however, these two requirements may not be compatible.

## History of epidemiological modelling

The idea of invisible living creatures as agents of disease goes back at least to the writings of Aristotle (384 BC-322 BC). It developed as a theory in the 16th century. The existence of microorganisms was demonstrated by Leeuwenhoek (1632-1723), with the aid of the first microscopes. The first expression of the germ theory of disease by Jacob Henle (1809-1885) came in 1840 and was developed by Robert Koch (1843-1910), Joseph Lister (1827-1912), and Louis Pasteur (1827-1875) in the latter part of the nineteenth century and the early part of the twentieth century.

The mechanism of transmission of infections is now known for most diseases. Generally, diseases transmitted by viral agents, such as influenza, measles, rubella (German measles) and chicken pox, confer immunity against reinfection, while diseases transmitted by bacteria, such as tuberculosis, meningitis and gonorrhea, confer no immunity against reinfection. Other diseases, such as malaria, are transmitted not directly from human to human but by vectors, agents (usually insects) that are infected by humans and subsequently transmit the disease back to humans.

The first mathematical model in epidemiology was the work of Daniel Bernoulli [4] on the effect of variolation against smallpox in increasing life expectancy. His work contained the idea of differential mortality to estimate the rate of deaths attributable to a given disease, a method which has been used to estimate disease death-rates of past epidemics, such as the 1918 influenza pandemic.

The foundations of mathematical epidemiology were laid in the late nineteenth and early twentieth centuries by public-health physicians and biological scientists such as P.D. En'ko [5], W.H. Hamer [6], J. Brownlee [7], Sir R.A. Ross [8], A.G. McKendrick and W.O. Kermack [3,9,10]. En'ko developed a discrete chain binomial model for the spread of infection in a susceptible population in 1889 [11].

Two of the landmarks in the development of mathematical epidemiology, illustrating the dichotomy between models for a specific disease and models for general classes of diseases, are the work of Ross on malaria, and the epidemic models of Kermack and McKendrick for classes of disease. Ross showed that malaria was transmitted through mosquitos and developed a model to describe the spread of malaria [8]. He then deduced from this model that reducing the mosquito population could control malaria in a region. This model was probably the first example of the threshold concept, which has been central in epidemiology ever since. The idea is that most mathematical epidemic models, including those that include a high degree of heterogeneity, usually exhibit "threshold" behaviour. In epidemiological terms, these can be stated as follows: *If the average number of secondary infections caused by a single infective introduced into a wholly susceptible population is less than one a disease will die out, while if it exceeds one there will be an epidemic*. This broad principle, consistent with observations and quantified via epidemiological models, has been used regularly to estimate the effectiveness of vaccination policies and the likelihood that a disease may be eliminated or eradicated. Hence, even if it is not possible to verify hypotheses accurately, agreement with hypotheses of a qualitative nature is often valuable.

The threshold principle is described quantitatively by the idea that the average number of secondary infections caused by an average infective is known as the basic reproduction number or basic reproductive ratio and denoted by ℛ_0 _[12]. It is a basic concept in mathematical epidemiology, derived originally from theoretical modelling considerations and then verified in observations. Its calculation for a given model and its estimation from observations are central in the analysis of models and the interpretation of data. If the basic reproduction number is less than one, then the infection dies out; if it exceeds one, then the infection persists.

The concept of the basic reproduction number was extended greatly in the work of Kermack and McKendrick [3,9,10] on general compartmental models, both for diseases in which recovery from infection conferred immunity against reinfection - commonly the case for diseases spread by viral infections - and for diseases in which recovered individuals are susceptible to reinfection, as is common for diseases transmitted by bacterial agents and for sexually transmitted diseases. What are usually described as the Kermack-McKendrick models are actually very special cases of the models in these papers, which included infectivity depending on age of infection and temporary immunity [13]. An offshoot of this work was the attempt by Soper [14] to explain the oscillations that had been observed in measles prevalence in many places. While Soper's explanation was flawed, it led to many other attempts to describe plausible models for measles [15-19] to explain the observed oscillations.

After the work of Kermack and McKendrick, there were many extensions of the basic models. The book by Bailey [20] describes many of the extensions that had been made up to the time of its publication in 1957; further updates during the next twenty years include [21-24], as well as the second edition of Bailey's book in 1975 [25]. Some of these refinements were made to give more realistic descriptions of microparasitic diseases by adding compartments. One such refinement was the incorporation of an exposed (latent) period, a time during which members of a population who have been infected do not pass on the infection to others. Another refinement was to models with temporary immunity against reinfection or to the assumption of a sequence of removed stages [26].

Another important aspect has been the question of a suitable representation for the rate of transmission of infection from an infective to a susceptible individual. The earliest models assumed a mass-action incidence law, possibly suggested by Hamer [6] and certainly present in the Ross malaria model. This law assumes that the average number of contacts sufficient to produce infection per individual in unit time is proportional to the population density. More recently, it has been noted that actual contact rates are not strongly dependent on population density. This suggests other forms for transmission rates, such as "standard incidence", in which it is assumed that the average number of contacts per individual in unit time is constant, or some sort of saturating contact rate [27,28]; see also [29-31] for recent references.

One main focus of mathematical epidemiology has been on the understanding and computation of the basic reproduction number in models with various kinds of heterogeneity. One useful abstract interpretation of the basic reproduction number is in terms of a "next generation" operator [32,33]. The addition of more structure in an epidemic model makes the interpretation and calculation of the basic reproduction number more difficult. The basic reproduction number, now universally denoted by ℛ_0_, is undoubtedly the most central idea in mathematical epidemiology, and its importance lies in its broad generality.

In an epidemic situation, where the time scale is short enough that there is are no births and no recruitment of new susceptibles into the population, and where recovery from disease confers full immunity against reinfection, the basic reproduction number marks a threshold between disappearance of the infection (basic reproduction number less than one) and the outbreak of an epidemic (basic reproduction number greater than one).

If there is a flow into the population of new susceptibles, the situation is different. If the basic reproduction number is less than one, there is a disease-free equilibrium and the infection dies out; if the basic reproduction number exceeds one, there is an *endemic *equilibrium and the disease may remain in the population. Some of the Kermack-McKendrick models [3,9,10] included proportional birth and death rates, which could allow exponential population growth in the absence of disease. This direction has been followed more recently [34-36]. For highly endemic diseases in resource-constrained countries, this is a plausible assumption. The question of whether and how an infectious disease alters the pattern of population growth is of particular concern [37]. However, in countries with more resources, it is more natural to expect bounded population size, suggesting nonlinear demographics. A first step is to assume a balance between births and deaths, keeping a constant total population size [22,38,39]. However, if there are deaths due to disease or a disease-related reduction of births, then it is not possible to keep the total population size constant and it is natural to assume nonlinear demographics, with density-dependent birth rates. Some of the papers that have incorporated nonlinear demographics into epidemiological models include [40-42]. An important direction of generalization has been the addition of more heterogeneity of various kinds in models. One kind of heterogeneity is heterogeneity of behaviour and the possibility that different subsets of a population may mix with different frequencies. This idea has been especially important in the study of sexually transmitted diseases [43-48], a topic of continuing interest. An important aspect of sexually transmitted diseases is that there is often a "core" group of very highly active individuals who are responsible for most of the disease transmission; control efforts aimed at this core group are likely to be most effective for control. This was analyzed in models in [49,50] and the translation of this analysis into action has been effective, especially for gonorrhea.

Other heterogeneities are also important. Many "childhood" diseases are transmitted mainly in school between children of the same age. Because age groups may mix heterogeneously, it may be appropriate to include age structure in epidemiological models [24,34,36,51-55]. Threshold results can be established for the existence of endemic states [56,57]. The incorporation of age structure leads to possibilities of behaviour that are not possible without the age dependence, such as sustained oscillations [19,58,59]. However, there is no indication of period-doubling or chaotic behaviour unless seasonal variation of contacts is assumed [16,60]. Age structure is an important aspect in the transmission of childhood diseases [61], e.g., for pertussis [62], rubella [29] and varicella [63]; it must be included in models designed to suggest realistic vaccination strategies. Optimal ages of vaccination are considered in [64,65]. Age structure can also be incorporated as the time since becoming infected (age of infection) [66]. This is an important characteristic of HIV/AIDS models [67].

Spatial heterogeneity in disease models takes two forms. One is local, namely diffusion in space. An introduction to models for the spatial spread of epidemics may be found in [[68], Chapter 20]; other references are [34,51,69-71]. One characteristic feature of such models is the appearance of traveling waves, which have been observed frequently in the spread of epidemics through Europe from medieval times to the more recent studies of fox rabies [68,72]. The asymptotic speed of spread of disease is the minimum wave speed [73-79]. Models describing spatial spread and including age of infection are analyzed in [68,80,81].

A second form of spatial heterogeneity is related to travel. With the advent of intercontinental air travel, it is possible for diseases to move from one location to a completely separate location very rapidly; this has led to the study of metapopulation models or models with patchy environments and movement between patches [82-88].

For some diseases (e.g., Chagas' disease), the epidemiological unit may not be an individual, but the essential question may be whether a household is infected [89]; see also [90-92] for more recent stochastic models that include household structure.

General models for diseases with vector transmission [24,93,94] grew out of the Ross malaria model. Vector transmission means simply that infection goes back and forth between two populations rather than being transmitted directly. Vector models are also appropriate in the study of heterosexually transmitted diseases, where the two populations are the males and females of the same species.

Vertical transmission, where newborn members of a population are infected, is a feature of many diseases, such as HIV/AIDS and Chagas' disease. A thorough description of diseases and models that include vertical transmission may be found in [95].

The original compartmental models assumed the rate of movement out of a compartment to be proportional to the number of members in the compartment. This is equivalent to the assumption of an exponentially distributed time spent in the compartment and leads to an ordinary differential equation model. For many diseases, a fixed time in a compartment, corresponding to a differential-difference equation model, is more realistic [96-100]. This assumption can lead to new possibilities for qualitative behaviour of a model [26,40,101]. More generally, an arbitrary distribution of times spent in a compartment can be assumed (see [102] and the bibliographical remarks there), leading to an integral equation or integro-differential equation model. Studies of realistic distributions may be found in [103-106]. The qualitative analysis of such models leads to questions of the location of roots of a transcendental equation. One consequence of results for such models is the possibility of unstable equilibria and sustained oscillations for some parameter sets in epidemic models. As these oscillations may have large amplitude and long period, they would be very troublesome in disease management. There have been several surveys of results of this nature [101,107], and work in this area continues [108,109].

Continuing in the tradition of Ross, there have been studies that focussed on specific diseases; see, for example, [35,49,110-114]. These have probably been the results of most immediate interest to field epidemiologists. However, it should not be overlooked that some fundamental ideas of practical importance, such as the relation between mean age at infection and the basic reproduction number, the concept of herd immunity, and the formulation of immunization strategies, were developed from simple, general models. For example, from data on smallpox, an estimated ℛ_0 _can be used to show that an immunization coverage of 70-80% should be sufficient to eradicate the disease [[111], Table 5.1]. The eradication of smallpox, declared in 1980 [115], was achieved by worldwide vaccination, and was a triumph for public health. This description has been mostly about deterministic compartmental models, but stochastic models have also been important. We will not go into the description and development of such models here, but some useful references are [20,90-92,114,116-124].

Another, relatively recent, development in disease transmission modelling has been the use of network models and detailed study of the network of contacts of an individual. Again, we do not go into the description and development but merely cite references [125-131] for the theoretical background and [132-136] for some simulations using network models for predictions of influenza pandemics. There is still much to be done in validating the simulation results and relating them to the theory. The origins of the study of mathematical epidemiology come from outside mathematics. As the mathematical analysis of epidemiological models progressed, epidemiologists took less account of the contributions of mathematics to epidemiology and mathematicians have not always been responsive to the questions that concern epidemiologists. Epidemiology and mathematical epidemiology appear to have diverged, but currently there are serious attempts to improve communications.

Some of the references cited contain historical information about the development of epidemiological models. A good description of the history up to 1975 may be found in [25]. Another important source of information about mathematical epidemiology is [111], which includes both descriptions of the properties of many communicable diseases and mathematical models. However, a full, up-to-date history has yet to be written. We may hope that if mathematicians and epidemiologists can come together, a history written in a few years would be radically different from a history that might be written today.

## What has modelling accomplished?

We have already mentioned two of the most striking contributions of mathematical modelling to disease management: the control of malaria through control of mosquitoes [8] and the elimination of smallpox by a sufficiently high vaccination rate [115]. Sir R. A. Ross was awarded the second Nobel prize in Medicine in 1902 for his work, beginning in 1882, in which he established that malaria was spread through contacts between humans and mosquitoes. Even though this discovery was honoured in the medical community, his conclusion that control of mosquitoes would be an aid in controlling malaria was not accepted because it was felt that it would be impossible to rid a region of mosquitoes and keep it mosquito-free. Only after Ross described a mathematical model [8] indicating that it was not necessary to remove the entire mosquito population to control the disease was this strategy adopted, with great success. In fact, Ross's model proved to be such a robust description of malaria that it remained current for about 50 years until it was updated by MacDonald [137].

Vaccination for smallpox, the world's first vaccine, was begun in 1796 by Edward Jenner, who had observed that people who had been infected with cowpox did not get smallpox. The recognition, from a smallpox model involving herd immunity, that vaccination of 70 *- *80% of a population would eliminate smallpox, led to an eradication program by the World Health Organization beginning in 1967; the last case in the Americas was in 1971 and the last case worldwide was in Somalia in 1977 [8,115,138-140].

Measles is a childhood disease which is easily controlled by vaccination, but in many resource-constrained countries, few children are vaccianted against measles and there are a million deaths from smallpox worldwide. Models with age structure have compared a strategy of a single dose of vaccine to a two-dose strategy; epidemiologists have concluded that a two-dose strategy of doses at age 12 to 15 months and 4 to 6 years is more effective. However, herd immunity would require an immune fraction of at least 0.94. Since vaccine efficacy for measles is about 0.95, it is unlikely that this can be achieved [29,141]. Thus, elimination of measles is unlikely to be achievable.

Another example of an important contribution of mathematical modelling is the control of sexually transmitted diseases through concentration on the most active members of the population [49]. Others include the management of bovine hoof and mouth disease in Great Britain through a process of culling infected herds of cattle as suggested by models [142,143].

To epidemiologists, the measure of whether a disease outbreak has been controlled is whether the reproduction number has been reduced to a value less than one. During the SARS epidemic of 2002-2003, the estimation of ℛ_0 _was the focus of many studies [144-146] and, after the epidemic had passed, models to compare the contribution of contact tracing and quarantine of suspected cases with the contribution of diagnosed infectives were studied. The conclusion appears to be that isolation was more effective and much less costly, partly because fewer than 5% of the people identified by contact tracing developed disease [147]. However, if infectivity had developed before the appearance of symptoms, which is now considered not to have been the case for SARS, contact tracing would have been more useful. The lessons learned from SARS are being applied to planning for a possible influenza pandemic.

For most disease transmission models, the expected situation is that if the basic reproduction number is less than one, then there is a globally asymptotic disease-free equilibrium, while if the basic reproduction number exceeds one, there is an endemic equilibrium. There are some situations [148-154] in which there may be a backward bifurcation for some parameter values as the basic reproduction number passes through one. In such cases, there may be an endemic equilibrium when the basic reproduction number is less than one (ruling out global asymptotic stability of the disease-free equilibrium) with discontinuities in behaviour as the basic reproduction number changes. Such behaviour is very unsettling for disease management and it is important to know from models when it can occur so that control evaluation can allow for the possibility. It can arise if there are groups with different susceptibility to infection or different contact rates as in models with partially effective vaccination [44,45,148,155,156].

Expressions for the basic reproductive number for HIV in various populations have been used to test the possible effectiveness of vaccines that may provide temporary protection by reducing either HIV-infectiousness or susceptibility to HIV. Models are used to estimate how widespread a vaccination plan must be to prevent or reduce the spread of HIV.

## Challenges for the future

While there are many infectious diseases which may pose huge problems in the near future, perhaps the two of most current concern are pandemic influenza and HIV/AIDS. For both, there are serious logistical questions concerning the availability and distribution of resources for management. Some basic questions are:

• How large a supply of drugs and medicines is needed?

• How can the necessary drugs and medicines be distributed?

• What happens to management strategies if the supply is insufficient?

• What might be the effects of the development of drug-resistant strains of infection?

• Can social distancing initiatives be helpful in disease management?

For HIV, one of whose aspects is the variation of infectivity with time since infection, detailed models will require an understanding of the development of virus in a host; models will need to link immunology and the cell level with infection and the individual level. Another difficulty in understanding HIV is that HIV can be a dormant virus in immune cells. The study of HIV on a cell level is well under way, but there is much more to be done; some basic references are [157-162]. Another aspect of HIV is the recognition that transmission depends strongly on the heterogeneity of contacts. Because HIV/AIDS is a disease with complicated scientific properties, it is of great interest to theoretical modelers, and because HIV/AIDS is so widespread and devastating, it is of great interest to scientists and also to governments. It is reasonable to hope that sufficient funding for research and treatment may be forthcoming.

Mathematical modelling has been a vital link between mathematics and physics for many years. A correspondingly strong link between mathematics and epidemiology would lead to great progress in epidemiological modelling. Currently, the mathematical content in the undergraduate education of students in the biological sciences is increasing; this should prove to be of great value in strengthening the links between mathematics and biology.

## Competing interests

The author declares that they have no competing interests.
